# Potential of angiotensin II receptor blocker telmisartan in reducing mortality among hospitalized patients with COVID-19 compared with recommended drugs

**DOI:** 10.1038/s41421-022-00454-7

**Published:** 2022-09-09

**Authors:** Dengyuan Liu, Peng Wu, Wentao Gu, Cuihong Yang, Xinmeng Yang, Xingyu Deng, Jun Xu, Jingmei Jiang, Chengyu Jiang

**Affiliations:** 1 State Key Laboratory of Medical Molecular Biology, Department of Biochemistry, Institute of Basic Medical Sciences Chinese Academy of Medical Sciences, School of Basic Medicine Peking Union Medical College, Beijing, China; 2 Department of Epidemiology and Biostatistics, Institute of Basic Medical Sciences, Chinese Academy of Medical Sciences, School of Basic Medicine, Peking Union Medical College, Beijing, China; 3 Emergency Department, State Key Laboratory of Complex Severe and Rare Diseases, Peking Union Medical College Hospital, Chinese Academy of Medical Science and Peking Union Medical College, Beijing, China

**Keywords:** Mechanisms of disease, Bioinformatics

Dear Editor,

More than 2 years have passed since the World Health Organization (WHO) announced the global pandemic of coronavirus disease 2019 (COVID-19). Since then, hundreds of millions of people have been reported to be infected with severe acute respiratory syndrome coronavirus-2 (SARS-CoV-2) and millions of death worldwide^1^. Different guidelines or agencies have recommended a number of drugs to treat COVID-19, and many randomized controlled trials (RCTs) have been carried out. Although currently not recommended, RCTs of angiotensin II receptor blocker (ARB) drugs telmisartan and losartan have demonstrated a significant reduction in the mortality rate among hospitalized patients with COVID-19 and those in the intensive care unit (ICU), respectively. It is necessary to analyze and compare the efficacy of these ARBs with recommended drugs using updated trial data. To minimize the variability among trials, we included only RCTs registered in ClinicalTrials.gov of the U.S. National Institutes of Health; we excluded outpatient trials and trials using combinations of drugs.

We analyzed RCTs that compared drugs for the treatment of COVID-19 against placebo or standard care. Two ARBs, telmisartan and losartan, were included in the analysis. We also included 12 drugs recommended in the Chinese Clinical Guidance for COVID-19 Pneumonia Diagnosis and Treatment (hereinafter, China 9th Edition), the WHO Guidelines for Therapeutics and COVID-19, or those granted emergency use authorization by the U.S. Food and Drug Administration (FDA) and European Medicines Agency (EMA). We searched ClinicalTrials.gov for relevant published and unpublished trials through May 23, 2022. Two reviewers independently extracted the data and assessed the trial methodology. The search strategy and the entire screening process are presented in Supplementary Fig. S1 and Table S1, and detailed information on eligible studies is presented in Supplementary Table S2.

To obtain a complete picture of the efficacy of the included drugs, the outcomes covered all-cause short-term and long-term mortality, including all-cause 5–8-, 14/15-, 21-, 25-, 28/30-, 35-, 45-, 60-, 70-, and 90-day mortality. We used risk ratios (RRs) and 95% confidence intervals (CIs) to assess the effect sizes in single or multiple trials (pooled in meta-analysis) for a certain drug on a specified outcome. The random-effects DerSimonian–Laird model was used for meta-analysis when needed, considering that some studies have large variation between them. Subgroup analyses were also performed with available data of patients in the ICU, those receiving invasive mechanical ventilation, and those who received oxygen only. The meta-analysis was performed in Stata 15 (StataCorp LLC, College Station, TX, USA) and forest plots were created with R 4.1.3 using the forestplot package (The R Project for Statistical Computing, Vienna, Austria).

We included 54 RCTs enrolling a total of 63,969 participants. Most trials focused on drug effectiveness with respect to 28/30-day mortality; the results of overall pooled analyses were presented in Fig. 1a. Compared with the control group, four drugs showed statistically significant results confirming the effects of intervention. These were telmisartan (no recommendation), which showed an 81% reduction in the risk of mortality (RR 0.19, 95% CI 0.06–0.62); dexamethasone (recommended by China, WHO) with a 10% reduction (RR 0.90, 95% CI 0.82–0.97), baricitinib (recommended by FDA, WHO) with a 36% reduction (RR 0.64, 95% CI 0.50–0.81), and REGEN-COV (recommended by FDA, EMA, WHO) with an 18% reduction (RR 0.82, 95% CI 0.73–0.92) in the risk of mortality. None of the remaining trials showed evidence of a difference in comparison with control groups. We present all results of every single trial for a specific drug in Supplementary Fig. S2.

**Fig. 1:**
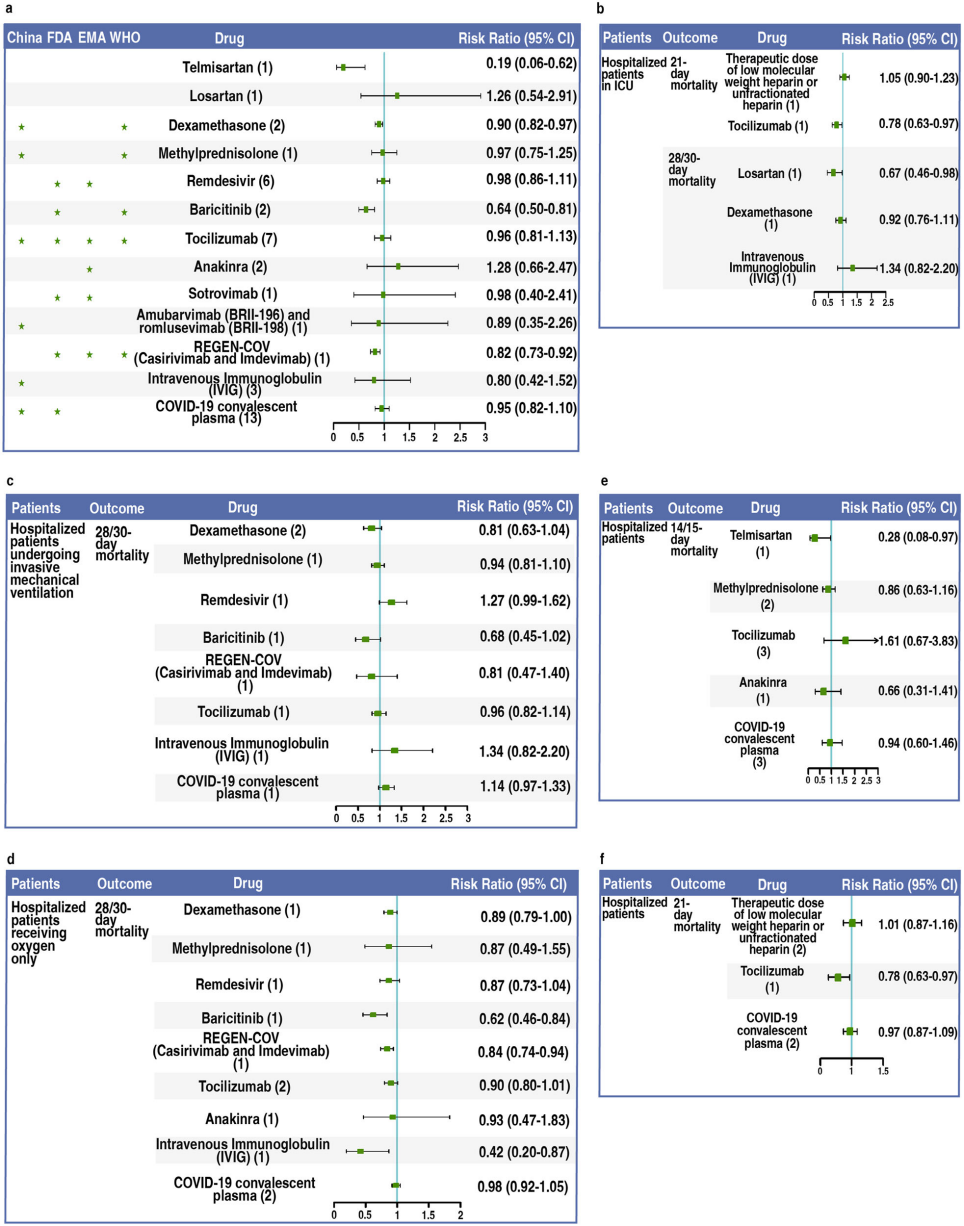
Outcome and efficacy of drugs in hospitalized patients with COVID-19. **a** Outcome (28/30-day mortality) and efficacy of telmisartan, losartan, and 11 drugs recommended by China, FDA, EMA and WHO. **b** Outcome (21/28/30-day mortality) and efficacy of drugs in hospitalized patients admitted to ICU. **c** Outcome (28/30-day mortality) and efficacy of drugs in hospitalized patients undergoing invasive mechanical ventilation. **d** Outcome (28/30-day mortality) and efficacy of drugs in hospitalized patients receiving oxygen only. **e** Outcome (14/15-day mortality) and efficacy of drugs in hospitalized patients. **f** Outcome (21-day mortality) and efficacy of drugs in hospitalized patients.

Figure 1b–d depicts the results of subgroup analysis, where at least one drug showed evidence of a beneficial effect in reducing mortality (except for Fig. 1c); the other results of subgroup analyses are presented in Supplementary Fig. S3. In terms of patients in the ICU (Fig. 1b), losartan exhibited a significant decrease in the risk of 28-day mortality (RR 0.67, 95% CI 0.46–0.98) among three intervention drugs; tocilizumab showed a significant decrease in the risk of 21-day mortality (RR 0.78, 95% CI 0.63–0.97). For the invasive mechanical ventilation subgroup (Fig. 1c), none of the eight drugs showed a significant difference with the control group in 28-day mortality. For patients who received oxygen only (Fig. 1d), among nine drugs, baricitinib demonstrated a significant 38% reduction (RR 0.62, 95% CI 0.46–0.84) and intravenous immunoglobulin showed a significant 58% reduction (RR 0.42, 95% CI 0.20–0.87) in 28-day mortality. Additionally, in terms of different periods of mortality, among five drugs with reported effects on 14/15-day mortality (Fig. 1e), only telmisartan demonstrated a significant 72% reduction (RR 0.28, 95% CI 0.08–0.97). Among three drugs with a reported effect of the intervention on 21-day mortality (Fig. 1f), only tocilizumab showed a significant 22% reduction (RR 0.78, 95% CI 0.63–0.97).

It is worth noting that among the drugs analyzed, six drugs (rows 1, 5, 7–9, and 13 in Supplementary Table S1) were investigated in 10 RCTs including outpatients. These trials were excluded because the outcome was usually composite (e.g., hospitalization or death), which made it difficult to evaluate the effect for a single endpoint. For the same reason, other recommended drugs (Paxlovid, molnupiravir, bamlanivimab, and etesevimab) investigated in seven RCTs involving only outpatients were also excluded.

Among the four drugs demonstrated to be effective (Fig. 1a), the ARB telmisartan may exhibit the highest reduction in mortality. Additionally, the ARBs losartan and telmisartan both demonstrated potentially good drug efficacy in subgroup analyses or in different outcome periods (Fig. 1b, e). ARBs are economical anti-hypertensive drugs that were first recommended to treat acute lung injury induced by SARS-CoV owing to imbalance of the renin–angiotensin system^2^. ARBs are reported to be effective in treating mouse acute lung injury induced by nanoparticles^3^ as well as infection with avian influenza A (H5N1 and H7N9) virus^4,5^. These studies suggest that ARBs might be a broad-spectrum treatment drug for patients with all-cause severe pneumonia in whom the renin–angiotensin system (angiotensin II is the biomarker) is altered, such as patients with COVID-19^6^. Unfortunately, most ARB trials were designed to include a mix of all individual ARB drugs, including telmisartan, losartan, valsartan, candesartan, irbesartan, eprosartan, olmesartan, and azilsartan, as well as a mix of all individual angiotensin-converting enzyme inhibitor (ACEI) drugs^7,8^. The results of these RCTs are not statistically significant^7,8^. In fact, the efficacies of ARBs in treating different diseases differ. The underlying mechanism could be their different binding affinities to angiotensin II receptor type 1 (AT1R)^9^ or an additional effect of inhibiting inflammation with some ARBs^10^. Telmisartan, which has the highest binding affinity with AT1R^9^, might be the most effective treatment in the ARB family for hospitalized patients with COVID-19. It is interesting that losartan, which demonstrates multiple anti-inflammatory effects independent of angiotensin receptor blockade^10^, was effective among patients in the ICU (Fig. 1b). Physicians are usually cautious about using anti-hypertensive drugs in intensive care. Our results show unusually promising outcomes among patients requiring critical care. Of note, a certain ARB may show different therapeutic effects in different subgroups or periods, and different ARBs may have inconsistent effects; therefore, we must interpret both with caution owing to the limitation that a small number of studies with small sample sizes were synthesized. However, promising therapeutic prospects were revealed. It should be emphasized that the aim of the present study was not to provide a definitive conclusion about the treatment effect of ARBs but rather to provide valuable guidance and promote further high-quality evidence for ARBs in future research. Taken together, our findings indicate that telmisartan might be recommended for hospitalized patients with COVID-19 and losartan might be recommended for patients in the ICU. Further studies and RCTs of telmisartan and losartan are necessary to confirm the treatment efficacy of these economical anti-hypertensive drugs for hospitalized patients with COVID-19 and those with all-cause severe pneumonia. These drugs might be potential candidates for use in future pandemics.

## Supplementary information

Supplementary Information
