# Supplementary material for: Potential of angiotensin II receptor blocker telmisartan in reducing mortality among hospitalized patients with COVID-19 compared with recommended drugs
Source: Cell Discov. 2022 Sep 9;8:91. doi: 10.1038/s41421-022-00454-7 (PMC9462647; doi:10.1038/s41421-022-00454-7)
Supplement: Supplementary file 1 — Supplementary Information [file 41421_2022_454_MOESM1_ESM.pdf]

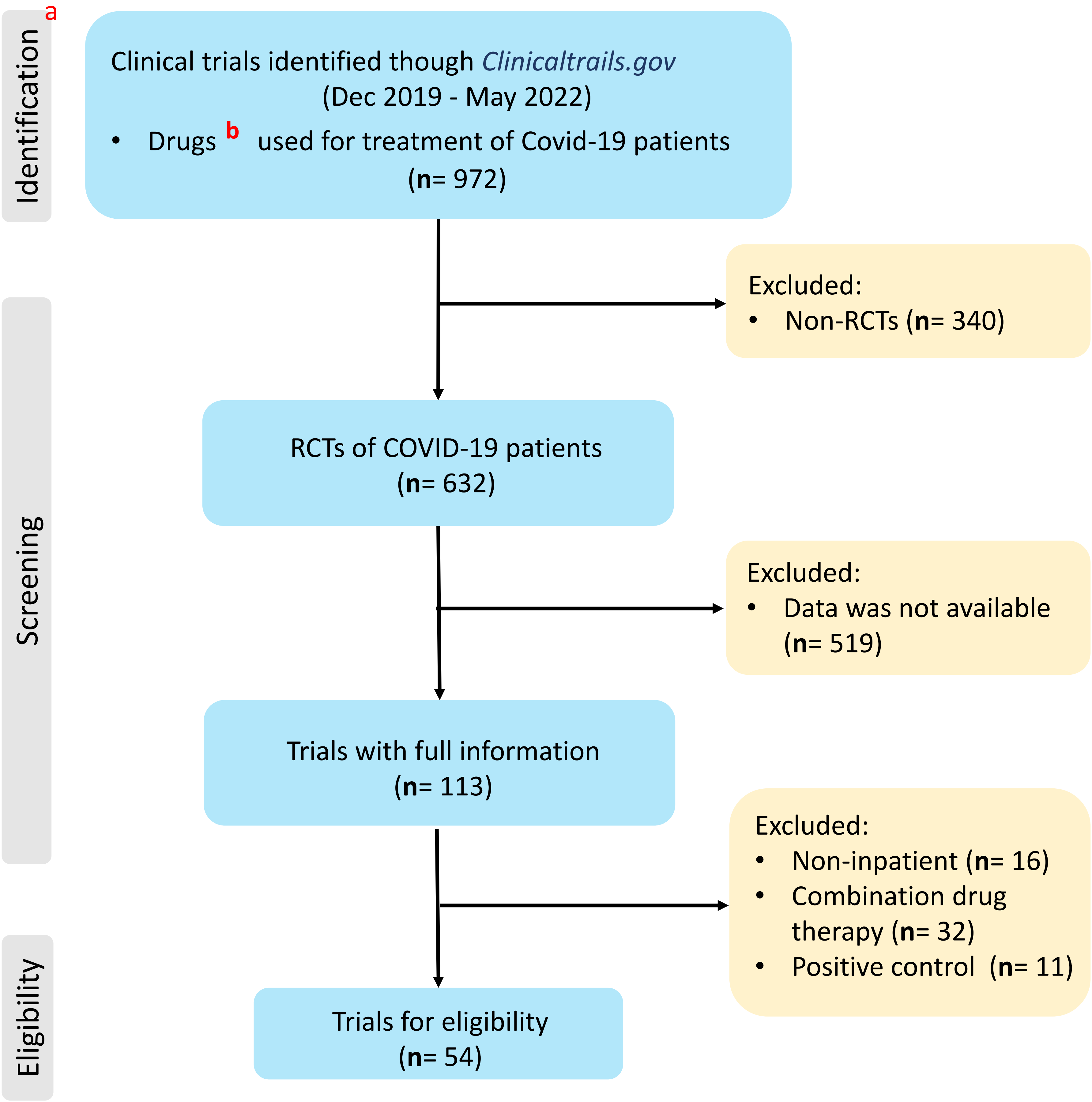

a Two authors (C.Y. and X.Y.) independently screened relevant trials.

b Drugs used for treatment include Angiotensin receptor blockers (ARB), as well as drugs recommended/emergency use authorized by the Chinese Clinical Guidance for COVID-19 Pneumonia Diagnosis and Treatment (China 9th Edition), the U.S. Food and Drug Administration (FDA), the European Medicines Agency (EMA) and those strongly recommended by the Guideline Therapeutics and COVID-19 of World Health Organization (WHO).

Supplementary Fig. S1 Flow diagram of study selection.

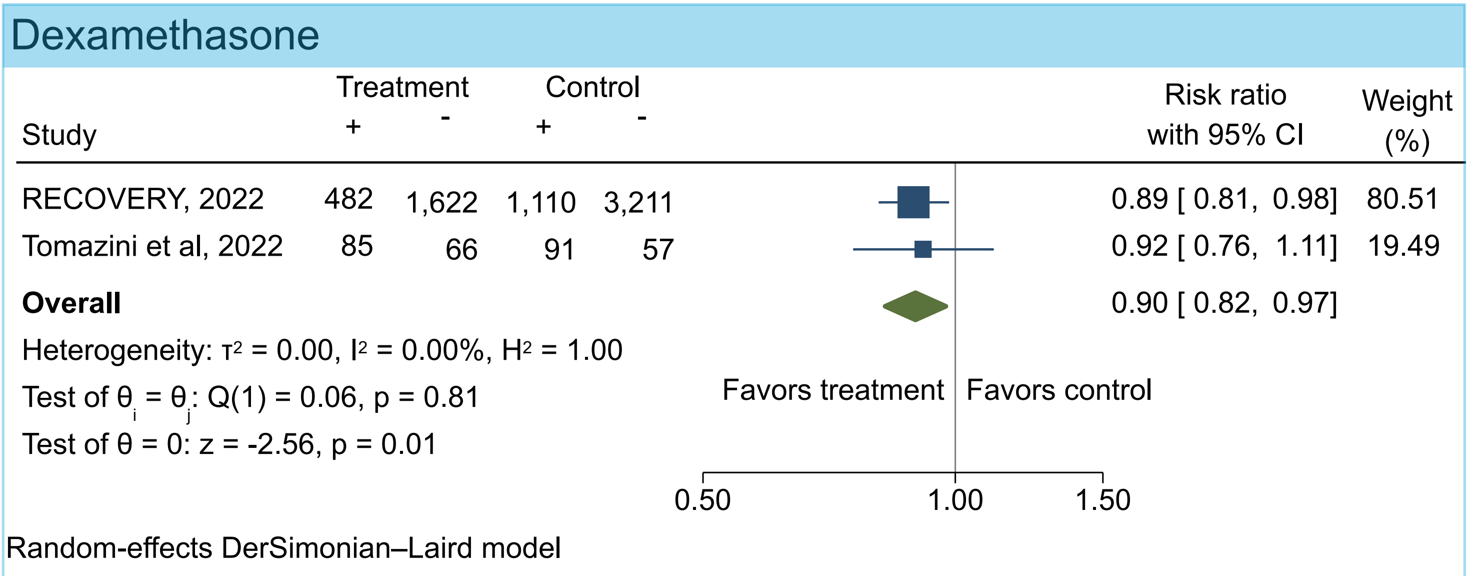
Dexamethasone
Study
Treatment
+ -
Control
+ -
Risk ratio with 95% CI
Weight (%)
RECOVERY, 2022 482 1,622 1,110 3,211 0.89 [0.81, 0.98] 80.51
Tomazini et al, 2022 85 66 91 57 0.92 [0.76, 1.11] 19.49
Overall 0.90 [0.82, 0.97]
Heterogeneity: τ² = 0.00, I² = 0.00%, H² = 1.00
Test of θi = θj: Q(1) = 0.06, p = 0.81
Test of θ = 0: z = -2.56, p = 0.01
Favors treatment
Favors control
0.50
1.00
1.50
Random-effects DerSimonian–Laird model

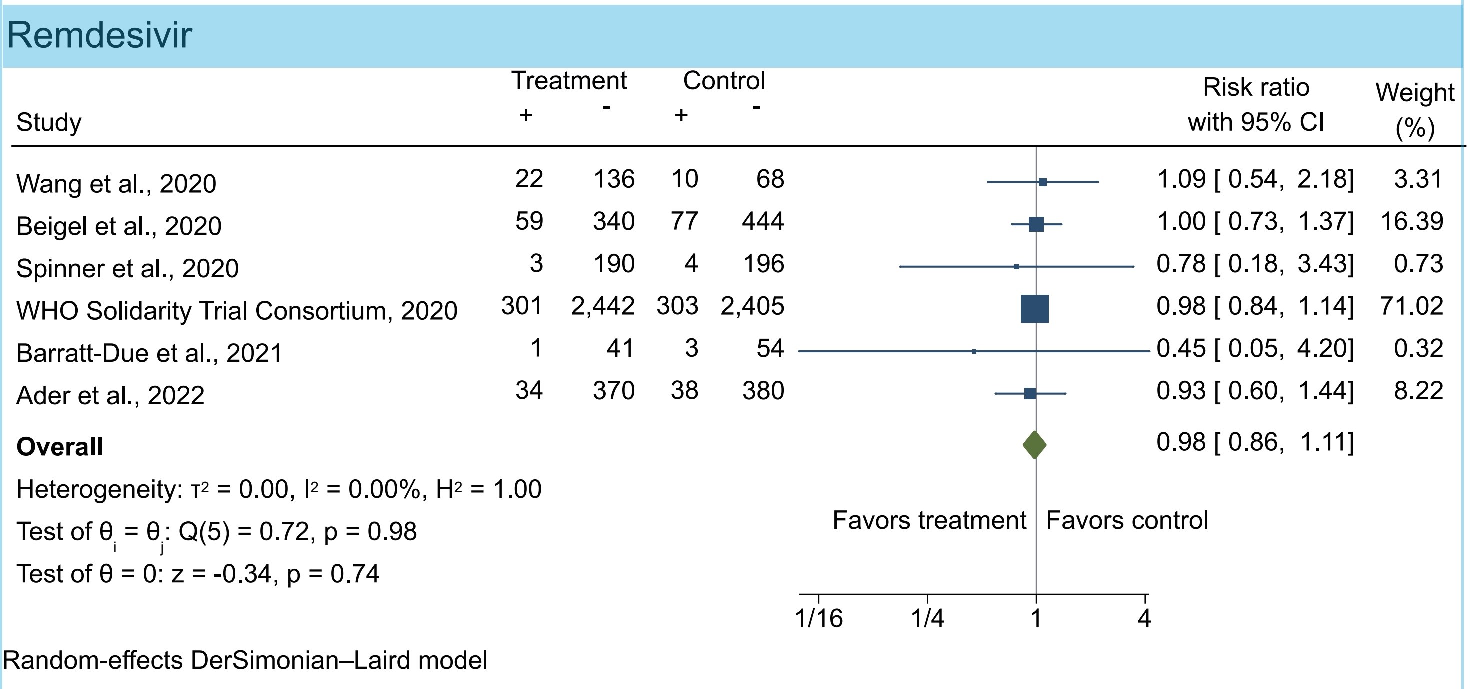
Remdesivir
Study
Treatment
+ -
Control
+ -
Risk ratio with 95% CI
Weight (%)
Wang et al., 2020 22 136 10 68 1.09 [0.54, 2.18] 3.31
Beigel et al., 2020 59 340 77 444 1.00 [0.73, 1.37] 16.39
Spinner et al., 2020 3 190 4 196 0.78 [0.18, 3.43] 0.73
WHO Solidarity Trial Consortium, 2020 301 2,442 303 2,405 0.98 [0.84, 1.14] 71.02
Barratt-Due et al., 2021 1 41 3 54 0.45 [0.05, 4.20] 0.32
Ader et al., 2022 34 370 38 380 0.93 [0.60, 1.44] 8.22
Overall 0.98 [0.86, 1.11]
Heterogeneity: τ² = 0.00, I² = 0.00%, H² = 1.00
Test of θi = θj: Q(5) = 0.72, p = 0.98
Test of θ = 0: z = -0.34, p = 0.74
Favors treatment
Favors control
1/16
1/4
1
4
Random-effects DerSimonian–Laird model

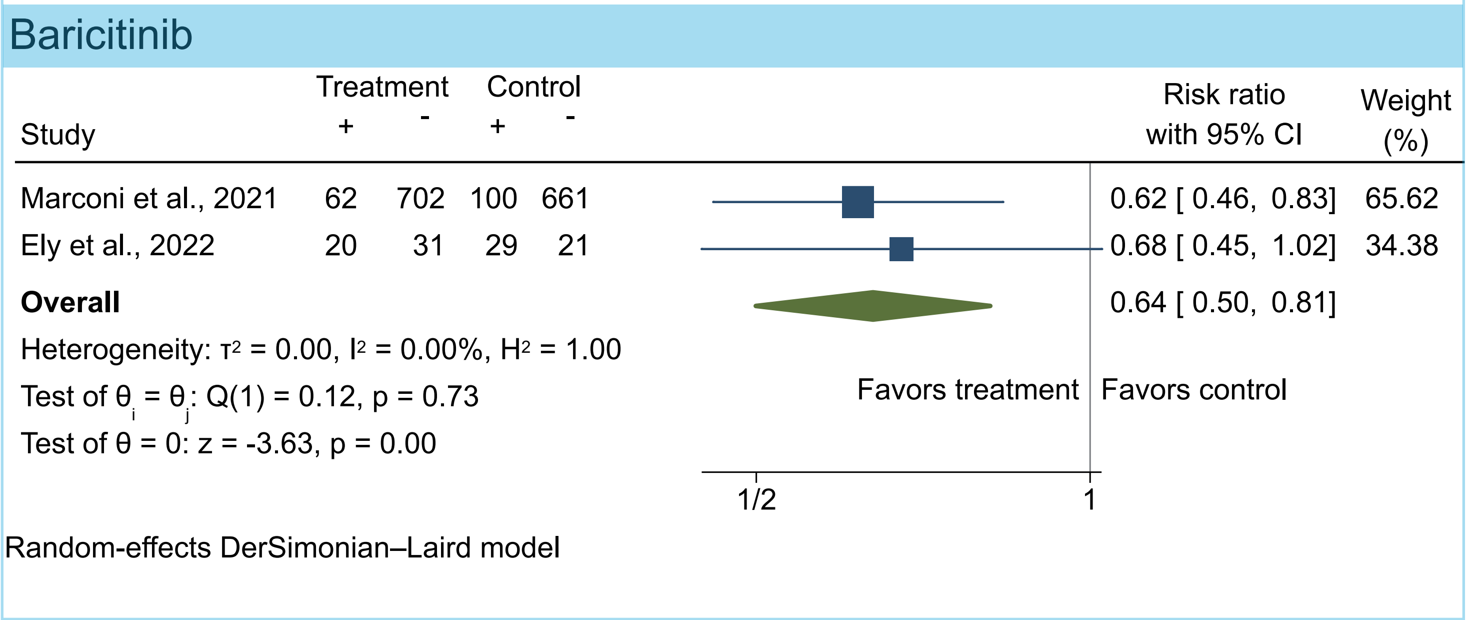
Baricitinib
Study
Treatment
+ -
Control
+ -
Risk ratio with 95% CI
Weight (%)
Marconi et al., 2021 62 702 100 661 0.62 [0.46, 0.83] 65.62
Ely et al., 2022 20 31 29 21 0.68 [0.45, 1.02] 34.38
Overall 0.64 [0.50, 0.81]
Heterogeneity: τ² = 0.00, I² = 0.00%, H² = 1.00
Test of θi = θj: Q(1) = 0.12, p = 0.73
Test of θ = 0: z = -3.63, p = 0.00
Favors treatment
Favors control
1/2
1
Random-effects DerSimonian–Laird model

Tocilizumab

Study	Treatment +	Treatment -	Control +	Control -	Risk ratio with 95% CI	Weight (%)
Azienda Unità Sanitaria Locale Reggio Emilia, 2020	2	58	1	62	2.10 [0.20, 22.56]	0.48
Hermine et al, 2020	7	56	8	59	0.93 [0.36, 2.42]	2.91
Salama et al, 2020	26	223	11	117	1.22 [0.62, 2.38]	5.68
Stone et al, 2020	9	152	3	78	1.51 [0.42, 5.42]	1.64
RECOVERY, 2021	621	1,401	729	1,365	0.88 [0.81, 0.96]	71.87
Rosas et al, 2021	58	236	28	116	1.01 [0.68, 1.52]	14.11
Veiga et al, 2021	14	51	6	58	2.30 [0.94, 5.61]	3.31
**Overall**					0.96 [0.81, 1.13]	

Heterogeneity: $\tau^2 = 0.01$, $I^2 = 9.89\%$, $H^2 = 1.11$

Test of $\theta_i = \theta_j$: Q(6) = 6.66, p = 0.35

Test of θ = 0: z = -0.49, p = 0.63

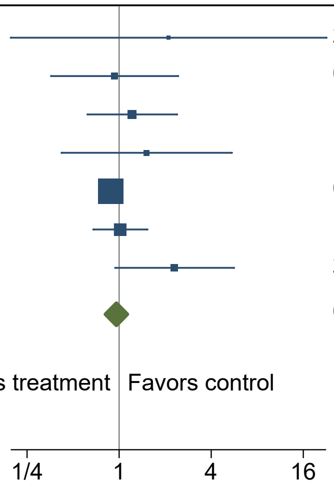

Random-effects DerSimonian–Laird model

Anakinra

Study	Treatment +	Treatment -	Control +	Control -	Risk ratio with 95% CI	Weight (%)
The CORIMUNO-19 Collaborative group, 2021	13	45	13	42	0.95 [0.48, 1.86]	56.13
Declercq et al., 2021	10	34	9	65	1.87 [0.82, 4.24]	43.87
**Overall**					1.28 [0.66, 2.47]	

Heterogeneity: $\tau^2 = 0.08$, $I^2 = 36.26\%$, $H^2 = 1.57$

Test of $\theta_i = \theta_j$: Q(1) = 1.57, p = 0.21

Test of θ = 0: z = 0.73, p = 0.47

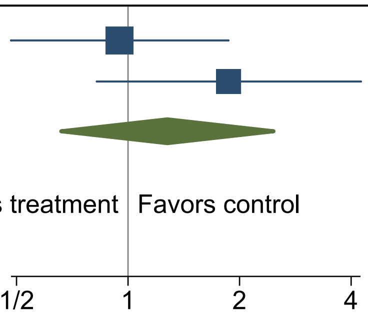

Random-effects DerSimonian–Laird model

Intravenous Immunoglobulin (IVIG)

Study	Treatment +	Treatment -	Control +	Control -	Risk ratio with 95% CI	Weight (%)
Ali et al., 2021	10	30	6	4	0.42 [0.20, 0.87]	29.31
The ITAC (INSIGHT 013) Study Group, 2022	18	277	22	262	0.79 [0.43, 1.44]	33.63
Mazeraud et al., 2022	24	45	20	57	1.34 [0.82, 2.20]	37.06
**Overall**					0.80 [0.42, 1.52]	

Heterogeneity: $\tau^2 = 0.23$, $I^2 = 70.68\%$, $H^2 = 3.41$

Test of $\theta_i = \theta_j$: Q(2) = 6.82, p = 0.03

Test of θ = 0: z = -0.69, p = 0.49

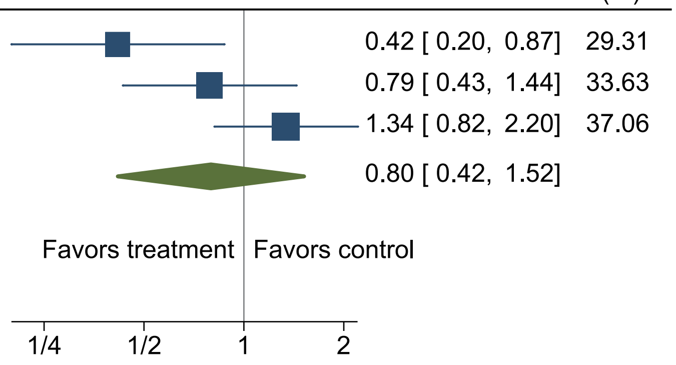

Random-effects DerSimonian–Laird model

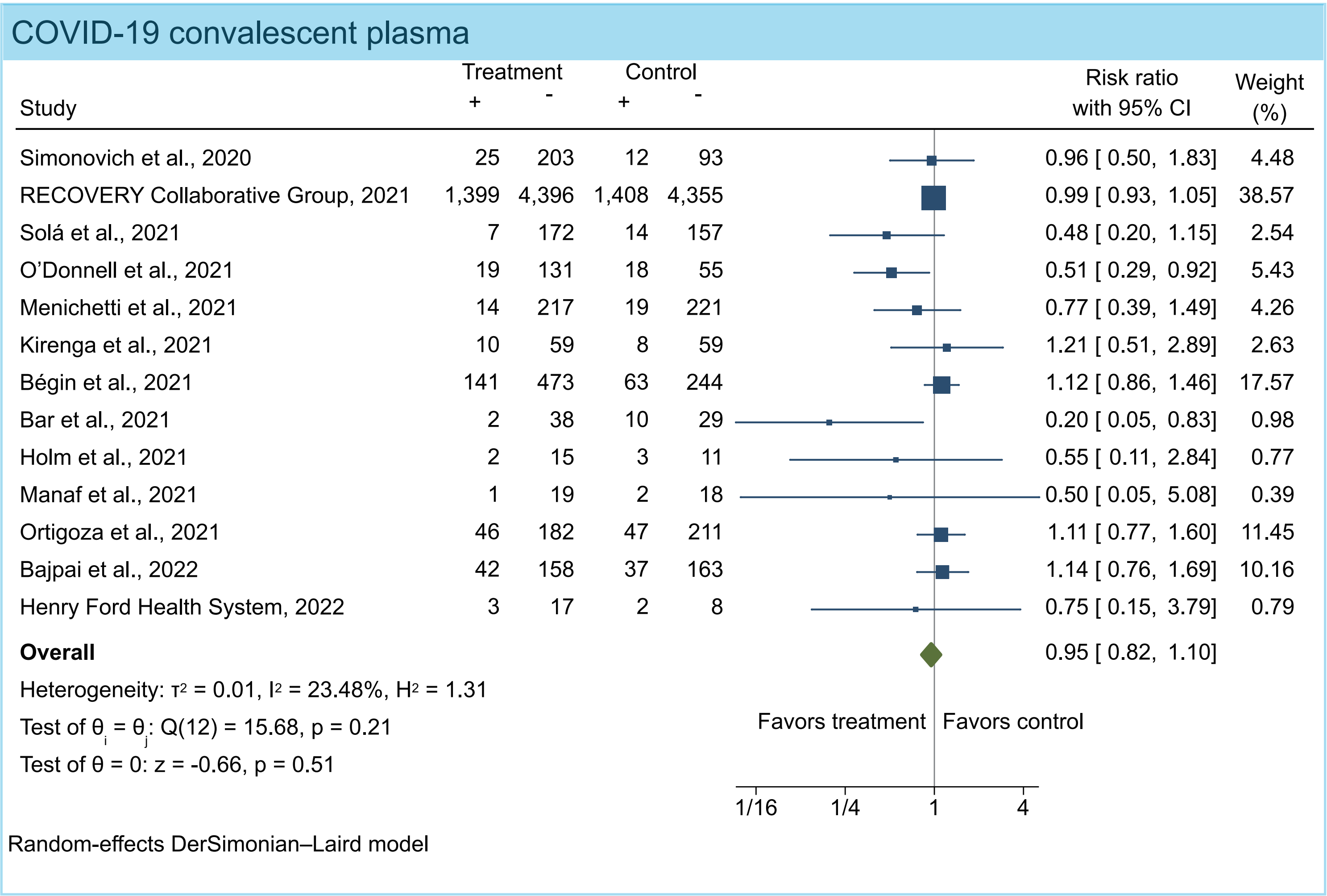

Supplementary Fig. S2 Outcome (28/30-day mortality) and efficacy of different drugs in hospitalized patients with Covid-19 using meta-analysis.

Patients	Outcome	Drug	Risk Ratio (95% CI)
Hospitalized patients	5–8-day mortality	Methylprednisolone (1)	0.7 (0.47-1.05)
		Tocilizumab (1)	4.92 (0.59-40.98)
		Amubarvimab (BRII-196) and romlusevimab (BRII-198) (1)	1.01 (0.06-15.95)
		COVID-19 convalescent plasma (1)	1.19 (0.69-2.06)
	25-day mortality	COVID-19 convalescent plasma (1)	0.50 (0.09-2.65)
	35-day mortality	COVID-19 convalescent plasma (1)	0.56 (0.26-1.22)
	45-day mortality	Losartan (1)	0.94 (0.06-13.68)
		Intravenous Immunoglobulin (IVIG) (1)	0.35 (0.04-3.06)
	60-day mortality	Remdesivir (1)	1.36 (0.29-6.39)
		Baricitinib (1)	0.73 (0.5-1.06)
		COVID-19 convalescent plasma (1)	0.63 (0.33-1.22)
	70-day mortality	Anakinra (1)	3.50 (0.17-70.94)
	90-day mortality	Methylprednisolone (1)	1.10 (0.07-16.43)
		Amubarvimab (BRII-196) and romlusevimab (BRII-198) (1)	1.17 (0.57-2.38)
		Anakinra (1)	1.01 (0.56-1.84)
		COVID-19 convalescent plasma (1)	1.13 (0.08-16.55)
Hospitalized patients undergoing invasive mechanical ventilation	60-day mortality	Baricitinib (1)	0.73 (0.50-1.06)
Hospitalized patients receiving oxygen only	14-day mortality	Tocilizumab (1)	1.24 (0.44-3.49)
		Anakinra (1)	0.65 (0.30-1.39)
		COVID-19 convalescent plasma (1)	1.26 (0.73-2.17)
	45-day mortality	Intravenous Immunoglobulin (IVIG) (1)	0.35 (0.04-3.06)
	90-day mortality	Anakinra (1)	0.99 (0.55-1.81)

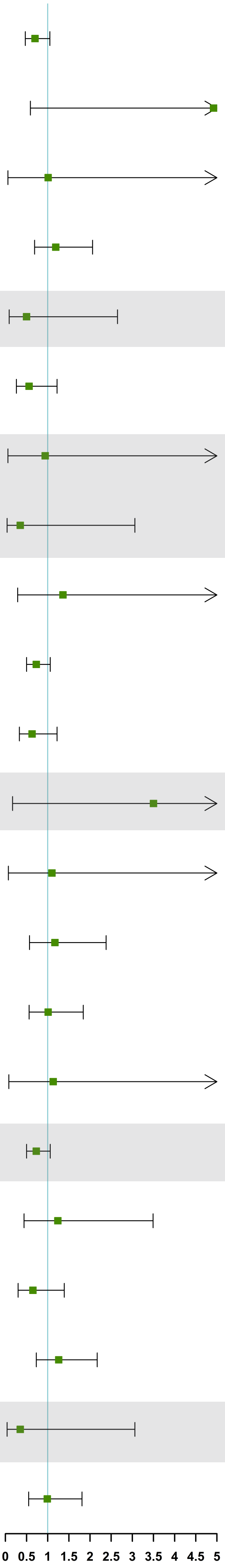

Supplementary Fig. S3 Evidence of absence effect in hospitalized patients with Covid-19.

Supplementary Table S1. Search strategies.

Drug name	Other names	Condition or disease in clinicaltrials.gov	Terms in clinicaltrials.gov	Search results
ARB	Losartan, Telmisartan, Valsartan, Telsartan, Candesartan, Irbesartan, Eprosartan, Olmesartan, Azilsartan	COVID-19	Losartan **OR** Telmisartan **OR** Valsartan **OR** Telsartan **OR** Candesartan **OR** Irbesartan **OR** Eprosartan **OR** Olmesartan **OR** Azilsartan	**31** Studies found for: Losartan OR Telmisartan OR Valsartan OR Telsartan OR Candesartan OR Irbesartan OR Eprosartan OR Olmesartan OR Azilsartan | COVID-19
Dexamethasone	NA[a]	COVID-19	Dexamethasone	**105** Studies found for: Dexamethasone | COVID-19
Methylprednisolone	NA	COVID-19	Methylprednisolone	**73** Studies found for: Methylprednisolone | COVID-19
Therapeutic dose of low molecular weight heparin or unfractionated heparin	NA	COVID-19	Therapeutic dose of low molecular weight heparin or unfractionated heparin **OR** heparin	**112** Studies found for: Therapeutic dose of low molecular weight heparin or unfractionated heparin OR heparin | COVID-19
Remdesivir	GS-5734, Veklury	COVID-19	Remdesivir **OR** GS-5734 **OR** Veklury	**136** Studies found for: Remdesivir OR GS-5734 OR Veklury | COVID-19
Baricitinib	Olumiant, LY3009104	COVID-19	Baricitinib **OR** Olumiant **OR** LY3009104	**27** Studies found for: Baricitinib | COVID-19
Sotrovimab	VIR-7831, GSK4182136, Xevudy	COVID-19	Sotrovimab **OR** VIR-7831 **OR** GSK4182136 **OR** Xevudy	**24** Studies found for: Sotrovimab OR VIR-7831 OR GSK4182136 OR Xevudy | COVID-19
Casirivimab/Imdevimab	REGEN-COV, Ronapreve,REGN10933/ REGN10987	COVID-19	Casirivimab **OR** Imdevimab **OR** REGEN-COV **OR** Ronapreve OR REGN10933 OR REGN10987	**26** Studies found for: Casirivimab OR Imdevimab OR REGEN-COV OR Ronapreve OR REGN10933 OR REGN1098 | COVID-19
Amubarvimab/romlusevimab	BRII-196 /BRII-198	COVID-19	Amubarvimab **OR** romlusevimab **OR** BRII—196 **OR** BRII—198	**7** Studies found for: Amubarvimab OR romlusevimab OR BRII-196 OR BRII-198 | COVID-19
Tocilizumab	NA	COVID-19	Tocilizumab **OR** RoActemra	**101** Studies found for: Tocilizumab OR RoActemra | COVID-19
Anakinra	Kineret	COVID-19	Anakinra **OR** Kineret	**40** Studies found for: Anakinra OR Kineret | COVID-19
Intravenous Immunoglobulin	IVIG	COVID-19	Intravenous Immunoglobulin **OR** IVIG	**89** Studies found for: Intravenous Immunoglobulin OR IVIG | COVID-19
COVID-19 convalescent plasma	NA	COVID-19	COVID-19 convalescent plasma	**196** Studies found for: COVID-19 convalescent plasma | COVID-19
Human COVID-19 immunoglobulin (Ph4) for intravenous injection	NA	COVID-19	Human COVID-19 immunoglobulin (Ph4) for intravenous injection	**2** Studies found for: Human COVID-19 immunoglobulin (Ph4) for intravenous injection | COVID-19

[a] NA means the drug has no other name

Supplementary Table S2. The basic characteristics of eligible studies.

NO	Study	ClinicalTrials.gov Identifier	Experimental group	Guideline[a]	Control group	Multicenter	Sample size	Availability of patient data			All-cause mortality	Published or Not
								ICU	Mechanical ventilation	Receiving oxygen only		
1	Puskarich et al., 2022	NCT04312009	Losartan	NA[b]	Placebo	Yes	205	Available	Unavailable	Unavailable	28d	Yes
2	Geriak et al., 2021	NCT04340557	Losartan	NA	Standard care	Yes	31	Unavailable	Unavailable	Unavailable	45d	Yes
3	Duarte et al., 2021	NCT04355936	Telmisartan	NA	Standard care	Yes	158	Unavailable	Unavailable	Unavailable	15d, 30d	Yes
4	Tomazini et al., 2022	NCT04327401	Dexamethasone	China, WHO	Standard care	Yes	299	Available	Available	Unavailable	28d	Yes
5	The RECOVERY Collaborative Group, 2020	NCT04381936	Dexamethasone	China, WHO	Standard care	Yes	6425	Unavailable	Available	Available	28d	Yes
6	Odeyemi et al., 2022	NCT03852537	Methylprednisolone	China, WHO	Standard care	No	41	Unavailable	Unavailable	Unavailable	90d	Yes
7	Tang et al., 2021	NCT04273321	Methylprednisolone	China, WHO	Placebo	Yes	86	Unavailable	Unavailable	Unavailable	14d	Yes
8	Christiane Maria Prado Jeronimo, 2020	NCT04343729	Methylprednisolone	China, WHO	Placebo	No	416	Unavailable	Available	Available	7d, 14d, 28d	Yes
9	The REMAP-CAP, ACTIV-4a, and ATTACC Investigators, 2021	NCT04372589	Therapeutic dose of low molecular weight heparin or unfractionated heparin	China	Standard care	Yes	1207	Available	Unavailable	Unavailable	21d	Yes
10	The ATTACC, ACTIV-4a, and REMAP-CAP Investigators, 2021	NCT04372589	Therapeutic dose of low molecular weight heparin or unfractionated heparin	China	Standard care	Yes	2244	Unavailable	Unavailable	Unavailable	21d	Yes
11	Barratt-Due et al., 2021	NCT04321616	Remdesivir	FDA, EMA	Standard care	Yes	366	Unavailable	Unavailable	Unavailable	28, 60d	Yes
12	Wang et al., 2020	NCT04257656	Remdesivir	FDA, EMA	Placebo	Yes	236	Unavailable	Unavailable	Unavailable	28d	Yes
13	Beigel et al., 2020	NCT04280705	Remdesivir	FDA, EMA	Placebo	Yes	1062	Unavailable	Unavailable	Unavailable	29d	Yes
14	Spinner et al., 2020	NCT04292730	Remdesivir	FDA, EMA	Standard care	Yes	584	Unavailable	Unavailable	Unavailable	28d	Yes
15	WHO Solidarity Trial Consortium, 2020	NCT04315948	Remdesivir	FDA, EMA	Standard care	Yes	11330	Unavailable	Available	Available	28d	Yes
16	Ader et al., 2022	NCT04315948	Remdesivir	FDA, EMA	Standard care	Yes	832	Unavailable	Unavailable	Unavailable	29d	Yes
17	Ely et al., 2022	NCT04421027	Baricitinib	FDA, WHO	Standard care	Yes	101	Unavailable	Available	Unavailable	28d, 60d	Yes
18	Marconi et al., 2021	NCT04421027	Baricitinib	FDA, WHO	Placebo	Yes	1525	Unavailable	Unavailable	Available	28d	Yes
19	ACTIV-3/Therapeutics for Inpatients with COVID-19 (TICO) Study Group, 2022	NCT04501978	Sotrovimab	FDA、EMA	Placebo	Yes	536	Unavailable	Unavailable	Unavailable	28d	Yes
20	The RECOVERY Collaborative Group, 2022	NCT04381936	REGEN-COV (Casirivimab /Imdevimab)	FDA、EMA, WHO	Standard care	Yes	9785	Unavailable	Available	Available	28d	Yes
21	ACTIV-3/Therapeutics for Inpatients with COVID-19 (TICO) Study Group, 2022	NCT04501978	Amubarvimab/romlusevimab (BRII-196 /BRII-198)	China	Placebo	Yes	536	Unavailable	Unavailable	Unavailable	5d, 28d, 90d	Yes

22	The RECOVERY Collaborative Group, 2021	NCT04381936	Tocilizumab	China，FDA，EMA, WHO	Standard care	No	4116	Unavailable	Available	Available	28d	Yes
23	Rosas et al., 2021	NCT04320615	Tocilizumab	China，FDA，EMA, WHO	Placebo	Yes	452	Unavailable	Unavailable	Unavailable	28d	Yes
24	Veiga et al., 2021	NCT04403685	Tocilizumab	China，FDA，EMA, WHO	Standard care	Yes	129	Unavailable	Unavailable	Unavailable	8d, 15d, 28d	Yes
25	Declercq et al., 2021	NCT04330638	Tocilizumab	China，FDA，EMA, WHO	Placebo	Yes	342	Unavailable	Unavailable	Unavailable	15d	Yes
26	The REMAP-CAP Investigators, 2021	NCT02735707	Tocilizumab	China，FDA，EMA, WHO	Standard care	Yes	895	Available	Unavailable	Unavailable	21d	Yes
27	Salama et al., 2020	NCT04372186	Tocilizumab	China，FDA，EMA, WHO	Standard care	Yes	388	Unavailable	Unavailable	Unavailable	28d	Yes
28	Stone et al., 2020	NCT04356937	Tocilizumab	China，FDA，EMA, WHO	Standard care	Yes	243	Unavailable	Unavailable	Unavailable	28d	Yes
29	Salvarani et al., 2020	NCT04346355	Tocilizumab	China，FDA，EMA, WHO	Standard care	Yes	126	Unavailable	Unavailable	Unavailable	30d	Yes
30	Hermine et al., 2020	NCT04331808	Tocilizumab	China，FDA，EMA, WHO	Standard care	Yes	131	Unavailable	Unavailable	Available	14d，28d	Yes
31	Swedish Orphan Biovitrum, 2022	NCT04324021	Anakinra	EMA	Standard care	Yes	16	Unavailable	Unavailable	Unavailable	70d	No
32	The CORIMUNO-19 Collaborative group,2021	NCT04341584	Anakinra	EMA	Standard care	Yes	116	Unavailable	Unavailable	Available	14d, 28d, 90d	Yes
33	Declercq et al., 2021	NCT04330638	Anakinra	EMA	Placebo	Yes	342	Unavailable	Unavailable	Unavailable	28d	Yes
34	The ITAC (INSIGHT 013) Study Group, 2022	NCT04546581	Intravenous Immunoglobulin (IVIG)	China	Placebo	Yes	579	Unavailable	Unavailable	Unavailable	28d	Yes
35	Mazeraud et al., 2022	NCT04350580	Intravenous Immunoglobulin (IVIG)	China	Placebo	Yes	146	Available	Available	Unavailable	28d	Yes
36	Sakoulas et al., 2021	NCT04411667	Intravenous Immunoglobulin (IVIG)	China	Standard care	Yes	33	Unavailable	Unavailable	Available	45d	No
37	Ali et al., 2021	NCT04521309	Intravenous Immunoglobulin (IVIG)	China	Standard care	No	50	Unavailable	Unavailable	Available	28d	Yes
38	Bajpai et al., 2022	NCT04425915	COVID-19 convalescent plasma	China，FDA	Standard care	Yes	400	Unavailable	Unavailable	Unavailable	7d, 28d	Yes
39	Henry Ford Health System, 2022	NCT04385199	COVID-19 convalescent plasma	China，FDA	Standard care	No	30	Unavailable	Unavailable	Unavailable	28d	No
40	The RECOVERY Collaborative Group, 2021	NCT04381936	COVID-19 convalescent plasma	China，FDA	Standard care	Yes	11558	Unavailable	Available	Available	28d	Yes
41	Avendaño-Solá et al., 2021	NCT04345523	COVID-19 convalescent plasma	China，FDA	Standard care	Yes	350	Unavailable	Unavailable	Unavailable	14d, 28d	Yes
42	Donnell et al., 2021	NCT04359810	COVID-19 convalescent plasma	China，FDA	Placebo	Yes	223	Unavailable	Unavailable	Unavailable	28d	Yes
43	Menichetti et al., 2021	NCT04716556	COVID-19 convalescent plasma	China，FDA	Standard care	Yes	487	Unavailable	Unavailable	Unavailable	30d	Yes
44	Libster et al., 2021	NCT04479163	COVID-19 convalescent plasma	China，FDA	Placebo	Yes	160	Unavailable	Unavailable	Unavailable	25d	Yes
45	Hsue et al., 2021	NCT04421404	COVID-19 convalescent plasma	China，FDA	Placebo	Yes	34	Unavailable	Unavailable	Unavailable	90d	No
46	Kirenga et al., 2021	NCT04542941	COVID-19 convalescent plasma	China，FDA	Standard care	No	136	Unavailable	Unavailable	Unavailable	28d	Yes

47	Bégin et al., 2021	NCT04348656	COVID-19 convalescent plasma	China，FDA	Standard care	Yes	940	Unavailable	Unavailable	Unavailable	30d	Yes
48	Writing Committee for the REMAP-CAP Investigators, 2021	NCT02735707	COVID-19 convalescent plasma	China，FDA	Placebo	Yes	2011	Unavailable	Unavailable	Unavailable	21d	Yes
49	Holm et al., 2021	NCT04600440	COVID-19 convalescent plasma	China，FDA	Standard care	Yes	33	Unavailable	Unavailable	Unavailable	28d	Yes
50	Ortigoza et al., 2021	NCT04364737	COVID-19 convalescent plasma	China，FDA	Placebo	Yes	941	Unavailable	Unavailable	Available	14d, 28d	Yes
51	Bar et al., 2021	NCT04397757	COVID-19 convalescent plasma	China，FDA	Standard care	Yes	79	Unavailable	Unavailable	Unavailable	14d, 28	Yes
52	Körper et al., 2021	NCT04433910	COVID-19 convalescent plasma	China，FDA	Standard care	Yes	105	Unavailable	Unavailable	Unavailable	21, 35, 60d	Yes
53	AlQahtani et al., 2021	NCT04356534	COVID-19 convalescent plasma	China，FDA	Standard care	No	40	Unavailable	Unavailable	Unavailable	28d	Yes
54	Simonovich et al., 2021	NCT04383535	COVID-19 convalescent plasma	China，FDA	Placebo	Yes	333	Unavailable	Unavailable	Unavailable	30d	Yes

[a] China: the Chinese Clinical Guidance for COVID-19 Pneumonia Diagnosis and Treatment (China 9th Edition); FDA: the U.S. Food and Drug Administration; EMA: the European Medicines Agency; WHO: the Guideline for Therapeutics and COVID-19 of World Health Organization (WHO).

[b] NA means the drug was not included in any guideline.
